# Case-control Analysis of *Clostridium difficile*—Associated Diarrhea on a Gynecologic Oncology Service

**DOI:** 10.1155/S1064744994000578

**Published:** 1994

**Authors:** Steven E. Waggoner, James Barter, Gregorio Delgado, Willard Barnes

**Affiliations:** 1 Department of Obstetrics and Gynecology Georgetown University Medical Center Washington, DC USA; 2 Department of Obstetrics and Gynecology MC 2050 University of Chicago 5841 South Maryland Avenue Chicago IL 60637 USA; 3 Department of Obstetrics and Gynecology Loyola University Medical Center 2160 South 1st Avenue Maywood IL 60153 USA

## Abstract

*Objective:* The incidence, morbidity, and risk factors associated with *Clostridium difficile*-associated diarrhea (CDAD) were studied in a group of gynecologic oncology patients.

*Methods:* A case-control analysis of gynecologic oncology patients with CDAD was carried out from August 1986 through January 1989 in a university medical center.

*Results:* One hundred twenty-three stool samples were tested for *C. difficile* using the CDT latex agglutination test (Marion Diagnostics, Kansas City, MO). Thirty episodes of CDAD developed in 23 patients. From August 1986 through July 1988, the incidence was stable at 1.5 episodes/100 admissions. From August 1988 through January 1989, the incidence increased to 9.9 episodes/100 admissions (*P* = 0.005). Compared with patients with nonspecific antibiotic-associated diarrhea, the study patients were hospitalized longer prior to the development of symptoms (mean 15.2 vs. 9.2 days, *P* = 0.006) and were admitted more frequently with diarrhea (37% vs. 11%, *P* = 0.015). The rates of surgery, chemotherapy, and radiation therapy were similar. Fever (57% vs. 14%, *P* < 0.001), abdominal pain (40% vs. 6%, *P* < 0.001), bloody stools (27% vs. 3%, *P* = 0.006), and leukocytosis (64% vs. 26%, *P* = 0.011) were more common among the study cases. The duration, indication, and number of antibiotics administered were similar, though once started, the mean time to symptoms was longer in the study cases (13.7 vs. 6.1 days, *P* = 0.004). Seven relapses, 1 death, and 1 unplanned colostomy occurred among women with CDAD.

*Conclusions: C. difficile* is a serious cause of nosocomial morbidity in gynecologic oncology patients. Diarrhea developing after antibiotic exposure is more likely to be associated with *C. difficile* in patients whose symptoms develop several days after completing antibiotics and in patients with a history of CDAD.


Infectious Diseases in Obstetrics and Gynecology 2:154–161 (1994)
© 1994 Wiley-Liss, Inc.

### Case-Control Analysis of *Clostridium difficile*–Associated Diarrhea on a Gynecologic Oncology Service

Steven E. Waggoner, James Barter, Gregorio Delgado, and Willard Barnes

*Department of Obstetrics and Gynecology, Georgetown University Medical Center, Washington, DC*

### Abstract

***Objective:*** The incidence, morbidity, and risk factors associated with ***Clostridium difficile***-associated diarrhea (CDAD) were studied in a group of gynecologic oncology patients.

***Methods:*** A case-control analysis of gynecologic oncology patients with CDAD was carried out from August 1986 through January 1989 in a university medical center.

***Results:*** One hundred twenty-three stool samples were tested for ***C. difficile*** using the CDT latex agglutination test (Marion Diagnostics, Kansas City, MO). Thirty episodes of CDAD developed in 23 patients. From August 1986 through July 1988, the incidence was stable at 1.5 episodes/100 admissions. From August 1988 through January 1989, the incidence increased to 9.9 episodes/100 admissions ($P = 0.005$). Compared with patients with nonspecific antibiotic-associated diarrhea, the study patients were hospitalized longer prior to the development of symptoms (mean 15.2 vs. 9.2 days, $P = 0.006$) and were admitted more frequently with diarrhea (37% vs. 11%, $P = 0.015$). The rates of surgery, chemotherapy, and radiation therapy were similar. Fever (57% vs. 14%, $P < 0.001$), abdominal pain (40% vs. 6%, $P < 0.001$), bloody stools (27% vs. 3%, $P = 0.006$), and leukocytosis (64% vs. 26%, $P = 0.011$) were more common among the study cases. The duration, indication, and number of antibiotics administered were similar, though once started, the mean time to symptoms was longer in the study cases (13.7 vs. 6.1 days, $P = 0.004$). Seven relapses, 1 death, and 1 unplanned colostomy occurred among women with CDAD.

***Conclusions:*** ***C. difficile*** is a serious cause of nosocomial morbidity in gynecologic oncology patients. Diarrhea developing after antibiotic exposure is more likely to be associated with ***C. difficile*** in patients whose symptoms develop several days after completing antibiotics and in patients with a history of CDAD. © 1994 Wiley-Liss, Inc.

Key words

*Clostridium difficile*, antibiotics, oncology, female cancers, nosocomial morbidity

*Clostridium difficile* is the pathogen most often responsible for nosocomially acquired diarrhea.[1] Most cases occur in individuals with a history of recent antibiotic use, and the organism or its toxins can be detected in the stool of 12–30% of such individuals.[2–4] The clinical manifestations of *C. difficile*-associated diarrhea (CDAD) are quite variable, running a spectrum from mild, nuisance diarrhea which resolves spontaneously to severe, occasionally lethal infections characterized by pseudomembranous colitis, toxic megacolon, or colonic perforation.[5,6] Hospital-based epidemics are well documented and reflect the frequent acquisition of the bacterium from other patients, staff, and the hospital environment.[7,8]

In addition to antibiotic use, the risk factors implicated in the development of CDAD include underlying malignancy, immunosuppression, in-

Address correspondence/reprint requests to Dr. Steven E. Waggoner, Department of Obstetrics and Gynecology, MC 2050, University of Chicago, 5841 South Maryland Avenue, Chicago, IL 60637.

Dr. Gregorio Delgado is now at the Department of Obstetrics and Gynecology, Loyola University Medical Center, 2160 South 1st Avenue, Maywood, IL 60153.

Received June 6, 1994
Accepted September 19, 1994

Clinical Study

**TABLE 1. Summary of data regarding patients and episodes of diarrhea**

| | Cases | Controls | Total |
|---|---|---|---|
| Episodes of diarrhea identified | 32 | 91 | 123 |
| Number reviewed | 32 | 60 | 92 |
| Number excluded | 2 | 24 | 26 |
| Reasons for exclusion | | | |
| No antibiotic exposure | 0 | 3 | 3 |
| Insufficient diarrhea | 2[a] | 12[b] | 14 |
| Diarrhea attributable to another cause | 0 | 9[c] | 9 |
| Episodes of diarrhea for analysis | 30 | 36 | 66 |
| Patients with a single episode of diarrhea | 15 | 25 | 40 |
| Patients with multiple episodes of diarrhea | 6 | 6 | 12 |
| All positive | 6 | | 6 |
| All negative | | 4 | 4 |
| Negative then positive | | 2 | 2 |
| Age in years (mean and range) | 61 (30–76) | 59 (29–87) | |
| Cancer present (%) | 93 | 97 | |

[a]Two patients finishing treatment for CDAD who symptomatically improved.
[b]Mainly includes stool samples collected for evaluation of fever without diarrhea being present.
[c]Six patients with radiation enteritis, 2 with short-bowel syndrome, and 1 with an enterovaginal fistula.

creasing age, female gender, prolonged or recurrent hospitalizations, and exposure to chemotherapy.[3,9–14] Most of these risk factors are present in women with gynecologic malignancies. A description of the impact of this infection among this particular patient population has not been previously reported, although several case-control studies on different patient populations have compared individuals with CDAD with individuals without diarrhea[12,15] or with diarrhea not associated with antibiotic use.[3] Furthermore, several studies describing the risks associated with antibiotic use have not discussed antibiotic exposure prior to the hospitalization when CDAD was detected.[14–16] Other reports, including those focusing on oncology patients, have been based on a relatively small number of cases.[9,10,17,18]

In early 1989, an increase in the incidence of CDAD was observed among patients on a gynecologic oncology service. This epidemic, which included 1 death from toxic megacolon, led to the present case-control study on the impact of this infection among these patients, particularly in comparison with the morbidity of nonspecific antibiotic-associated diarrhea.

### SUBJECTS AND METHODS

A database in the hospital's clinical laboratory was surveyed to identify the results of all stool specimens submitted for *C. difficile* analysis from patients hospitalized on the gynecologic oncology service from August 1986 through January 1989 (123 stool samples collected from 106 patients). The stool samples were collected primarily from patients with diarrhea and occasionally as part of a fever evaluation. The latex agglutination test CDT (Marion Diagnostics, Kansas City, MO) was used throughout the study for the detection of *C. difficile.* This assay detects a cell-wall antigen of *C. difficile* and is a more rapid and less expensive alternative to tissue-culture-based cytotoxicity assays.[19,20]

Thirty-two CDT-positive and 91 CDT-negative episodes of diarrhea were identified from the laboratory reports (Table 1). Hospital and clinic charts from all positive episodes were reviewed. Two CDT-negative episodes for each CDT-positive episode were matched by month of testing and also reviewed. The eligibility criteria for final analysis included a history of current or recent (within 8 weeks) antibiotic use and significant diarrhea ($\geq$3 liquid stools in 24 h) not attributable to another cause. The incidence of CDAD infection was calculated from the ratio of the number of episodes of CDAD to the number of surgical and medically related admissions to the gynecologic oncology service over 6-month periods.

### Statistical Analysis

The differences between means were analyzed with the Student's t-test. The differences in frequencies

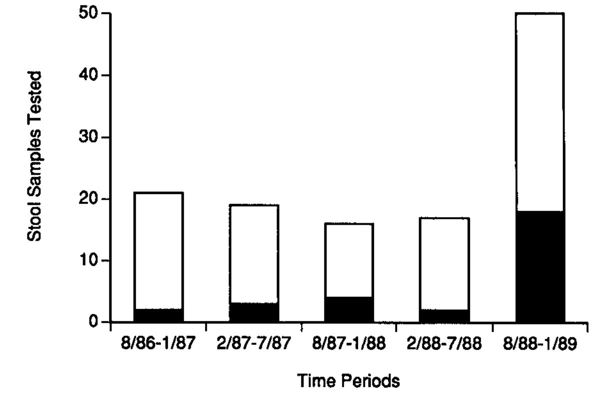

**Fig. 1. Prevalence of CDAD. Patients testing positive by the CDT assay are represented by the shaded areas and increased from 16% of total samples tested during the first 24 months of the study to 40% during the last 6 months (P = 0.0037).**

were evaluated with the Fisher exact test. Two-tailed tests were used for all comparisons.

### RESULTS

### Prevalence and Incidence

The stool samples were analyzed on 123 occasions over the 30-month study, representing a stool analysis of 12.6% of patients admitted to the gynecologic oncology service for surgery or medically related problems. The CDT assay was positive in 32/123 (26%) instances (Fig. 1). If one considers separately the periods from August 1986 through July 1988 and August 1988 through January 1989, the proportion of positive samples increased significantly from 12/73 (16%) instances to 20/50 (40%) instances ($P = 0.0037$). From August 1986 through July 1988, the 6-month incidence of CDAD was stable at 1.5 cases/100 admissions. From August 1988 through January 1989, the incidence increased sharply to 9.9 cases/100 admissions ($P = 0.005$, Fig. 2). No episodes of diarrhea were felt to be secondary to parasitic or other bacterial infections during the course of the study.

### Risk Factors (Table 2)

A similar proportion of the cases and the comparison group had been hospitalized in the 8 weeks preceding diarrhea; however, patients with CDAD spent a significantly greater number of days in the hospital over the 8 weeks prior to the onset of symptoms ($P = 0.006$). The cases were more commonly admitted with diarrhea as a chief complaint ($P = 0.015$), and each episode of CDAD which began at home had been preceded by 1 or more hospitalizations within the prior 8 weeks. Patients with CDAD were much less likely to have been admitted for a scheduled surgical procedure during the hospitalization when the diarrhea developed ($P = 0.002$). The relationship between surgery and the risk of CDAD was less significant ($P = 0.06$) if any surgical procedure in the 8 weeks preceding symptoms was considered. Exposure to chemotherapy in the 8 weeks preceding diarrhea as well as current or previous radiotherapy to the abdomen or pelvis was similar in the 2 groups.

### Morbidity (Table 3)

In comparison with patients with nonspecific diarrhea, the mean duration of diarrhea was significantly greater in patients with CDAD, as was the likelihood of fever, abdominal pain, and bloody stool ($P < 0.01$ for each symptom). When checked within 24 h of the CDT assay (cases, 25 instances; comparison groups, 23 instances), the mean white blood cell (WBC) count was significantly higher among patients with CDAD ($P = 0.018$). Among the cases, there was 1 death due to toxic megacolon and 1 patient required a colostomy for persistent lower intestinal bleeding secondary to pseudomembranous colitis.

### Antibiotic Profiles

Each patient received 1 or more courses of antibiotics in the 8 weeks preceding the onset of diarrhea. CDAD developed after exposure to a single antibiotic in 7 instances and after a single dose in 2 cases. The cumulative number of days of exposure to antibiotics over the 8 weeks preceding the onset of diarrhea did not differ significantly between the 2 groups ($12.7 \pm 11.8$ vs. $12.0 \pm 10.6$ days, $P = 0.82$). The average total number of different antibiotics given was also similar ($2.2 \pm 1.5$ vs. $2.5 \pm 1.5$, $P = 0.36$). The cases, however, more commonly received their most recent course of antibiotics prior to the hospitalization when the stool sample was sent for *C. difficile* analysis (19/30, 63% vs. 5/36, 14%, $P < 0.001$). Correspondingly, patients with nonspecific diarrhea were more

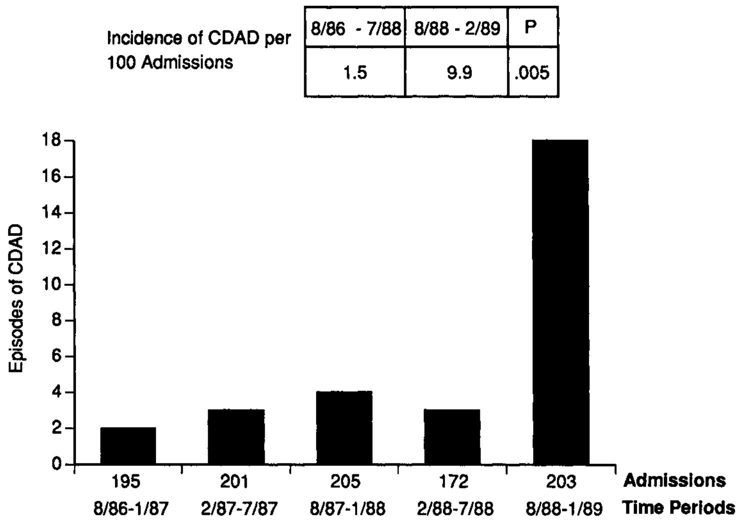

Fig. 2. Incidence of CDAD on the gynecologic oncology service from August 1986 through January 1989.

TABLE 2. Risk factors in the development of CDAC

| | Cases (N = 30) | Controls (N = 36) | *P* |
|---|---|---|---|
| Hospitalized within 8 weeks of symptoms (%) | 60 | 45 | 0.21 |
| Days hospitalized prior to symptoms (mean ± SD) | 15.2 ± 10.7 | 9.2 ± 5.2 | 0.006 |
| Admitted with diarrhea (%) | 36 | 11 | 0.015 |
| Admitted for surgery (%) | 30 | 78 | 0.002 |
| Surgery in the prior 8 weeks (%) | 57 | 78 | 0.06 |
| Recent chemotherapy (%) | 47 | 33 | 0.27 |
| Prior or current radiation therapy (%) | 37 | 26 | 0.10 |

TABLE 3. Morbidity associated with diarrhea

| | Cases (N = 30) | Controls (N = 36) | *P* |
|---|---|---|---|
| Days of diarrhea (mean ± SD) | 9.8 ± 6.3 | 2.1 ± 1.7 | <0.001 |
| Abdominal pain[a] (%) | 40 | 6 | <0.001 |
| Bloody stool[b] (%) | 27 | 3 | 0.006 |
| Fever ≥38.5°C (%) | 57 | 14 | <0.001 |
| WBC (× $10^9$/l, mean ± SD) | 14.7 ± 8.0 | 10.0 ± 4.8 | 0.018 |

[a]A patient's complaint or clinician's assessment of abdominal cramps, pain, or tenderness.
[b]Gross or occult evidence of blood in the stool.

likely to develop diarrhea while taking antibiotics or within 3 days of finishing antibiotics (26/36, 73% vs. 14/30, 47%, $P = 0.036$).

In 14 CDAD and 25 nonspecific episodes of diarrhea, a single course of antibiotics preceded the onset of symptoms (Table 4). For these patients, the number of different antibiotics given, total days of exposure, and indications for use were similar. The time interval from the onset of antibiotic exposure to the onset of symptoms differed significantly, however. Among patients with CDAD, the symptoms began an average of 13.7 days after receiving antibiotics in comparison with 6.1 days among the comparison group with nonspecific diarrhea ($P = 0.004$).

No antibiotic class was used with significantly greater frequency in the patients with CDAD compared with the comparison group, but exposure to

**TABLE 4. Episodes of diarrhea preceded by a single antibiotic regimen**

| | Cases (N = 14) | Controls (N = 25) | *P* |
|---|---|---|---|
| Total days of antibiotic exposure (mean ± SD) | 7.8 ± 6.7 | 7.2 ± 6.5 | 0.77 |
| Number of antibiotics (mean ± SD) | 1.9 ± 1.2 | 2.3 ± 1.3 | 0.35 |
| Days from onset of antibiotics to onset of symptoms (mean ± SD) | 13.7 ± 8.6 | 6.1 ± 2.9 | 0.004 |
| Indications for antibiotic use (%) | | | |
| Prophylactic | 50 | 40 | 0.39 |
| Empiric | 28 | 44 | 0.27 |
| Therapeutic | 21 | 16 | 0.23 |
| Specific antibiotic use (%) | | | |
| Cephalosporin | 86 | 72 | 0.28 |
| Penicillin | 21 | 36 | 0.28 |
| Metronidazole | 14 | 4 | 0.29 |
| Oral erythromycin-neomycin | 7 | 20 | 0.28 |
| Azactam | 14 | 16 | 0.64 |
| Imipenam | 7 | 0 | 0.36 |
| Clindamycin | 0 | 12 | 0.25 |
| Aminoglycoside | 7 | 40 | 0.030 |

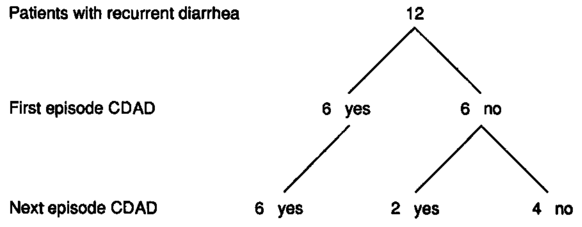

**Fig. 3. Influence of a prior episode of CDAD on recurrent diarrhea. Risk of recurrence with CDAD was highly associated ($P$ = 0.030) with a history of CDAD.**

aminoglycosides was significantly more common in patients without CDAD ($P$ = 0.030).

### Relapses

Twelve patients had more than 1 episode of diarrhea. Six had a history of CDAD; in each case, the recurrent diarrhea, 7 total episodes, was *C. difficile* positive. These patients represented 26% of the individuals who developed CDAD and accounted for 43% of the total episodes of CDAD. Six patients had a history of nonspecific diarrhea. Two of these individuals subsequently developed a single episode of CDAD, and the 4 other patients had 5 recurrent episodes of nonspecific diarrhea. Thus, a prior episode of CDAD was a significant risk factor in predicting whether recurrent diarrhea was also due to *C. difficile* ($P$ = 0.030, Fig. 3).

### Therapy

The therapy for CDAD included starting oral metronidazole or vancomycin and discontinuing other antibiotics if feasible. On the rare occasion that oral medication was not possible, IV metronidazole or vancomycin enemas were given. Medications contributing to bowel stasis were stopped, and fluid and electrolyte disturbances corrected. The duration of diarrhea, once the therapy had been started, averaged 4.8 days (range 1–18 days). Three of 7 relapses developed after initial treatment with metronidazole and 4 after vancomycin. All relapses responded to 1 or more additional courses of vancomycin.

### DISCUSSION

This study examines the risk factors, morbidity, antibiotic profiles, treatment response, and incidence of CDAD on a gynecologic oncology service. The cases and the comparison group were of the same sex, from the same ward, of similar age, and with a similar incidence of cancer. A single *C. difficile* assay was used throughout the study.

### Risk Factors

The most severe cases of antibiotic-associated diarrhea involve *C. difficile*, and toxin-producing *C. difficile* is rarely ever detected without a history of antibiotic use.[21,22] All of our cases had received 1 or more antibiotics within 8 weeks prior to the onset of symptoms, which supports the general belief that antibiotic use is the major risk factor in the etiology of CDAD.

*C. difficile* infections may occur as an epidemic in the hospital, and nosocomial transmission has been well described.[7,8,23] In the present series, a similar proportion of patients with CDAD and nonspecific diarrhea had been admitted to the hospital during the 8 weeks prior to the hospitalization when a stool sample was tested for *C. difficile*. Patients with CDAD, however, averaged more days in the hospital (mean 15.2 vs. 9.2 days, $P = 0.006$) which may reflect a greater opportunity of acquiring *C. difficile* from other patients, staff, or the hospital environment. The dramatic increase in the incidence of CDAD beginning in August 1988 lasted about 6 months before returning to baseline levels. The resolution of this epidemic coincided with an increased focus on the importance of handwashing and isolation of infected individuals.

Chemotherapeutic agents are occasionally considered risk factors in the development of CDAD,[13,24] though concurrent exposure to antibiotics is not uncommon.[25] In our study, all patients receiving chemotherapy who developed CDAD had also received 1 or more antibiotics during the same time period. Overall, 40% of the episodes of diarrhea in the study were preceded by chemotherapy in the previous 8 weeks, and no significant difference was noted between cases and the comparison group.

Radiation therapy was considered as possibly contributing to the risk of CDAD. Radiotherapy to the pelvis or abdomen often results in diarrhea during and after treatment. Antimotility agents, commonly used to control diarrhea, may increase the incidence of antibiotic-associated diarrhea and worsen its severity in established cases.[26] Forty-seven percent of cases and 33% of patients with nonspecific diarrhea had received radiotherapy to the abdomen or pelvis, an incidence not significantly different, therefore suggesting that radiation therapy was not an independent risk factor in our patient population.

Gastrointestinal surgery has been considered by some to be a risk factor for CDAD,[14,27] while others[25] have noted no apparent increase in CDAD after abdominal surgery in comparison with other surgical procedures. Controlling for horizontal transmission also reduces the apparent risk from surgery.[16] In our series, the cases were significantly less likely to have had surgery during the admission when stool samples were analyzed for *C. difficile*. This observation is biased by the greater likelihood of admission for medical problems, including diarrhea, among patients with CDAD when compared with patients with nonspecific diarrhea. As interactions between surgery and antibiotic exposure may occur several weeks prior to symptoms, a more appropriate analysis of the risk of surgery would compare the rates of surgery over the previous several weeks. When examined in this context, the percentage of cases having had surgery in the prior 8 weeks more closely approximates the incidence in the comparison group. In the present study, surgery to the small or large intestines was associated with CDAD diarrhea in 6 instances. Nonspecific diarrhea developed in 8 patients, which suggests that gastrointestinal surgery alone was not an independent risk factor in our patient population.

### Morbidity

Patients with CDAD can become quite ill and several series have described the morbidity associated with this illness.[12,25,28,29] Among cases in the present series, there was 1 death due to toxic megacolon and 1 unplanned colostomy for persistent colonic bleeding from pseudomembranous colitis. Additional morbidity among the patients with CDAD included fever (57%), abdominal pain or cramps (40%), bloody stool (27%), and leukocytosis (64%). These features were unusual in the comparison group. The duration of diarrhea, another manifestation of morbidity, was longer in the patients with CDAD. In 11 (37%) instances, patients treated with antibiotics were readmitted to the hospital with diarrhea or abdominal pain as a chief complaint and found to have *C. difficile*. One hundred eight patient-days were spent in the hospital as a result of this nosocomially acquired infection. In contrast, only 4 women with nonspecific diarrhea were admitted with diarrhea as a presenting complaint, and in no instance was diarrhea the primary complaint.

### Antibiotic Profiles

The duration and number of antibiotics have been considered by some to increase the risk of developing CDAD,[14,15] while others have not confirmed this.[10,12] Among our patients, there was no difference between cases and the comparison group in the duration of antibiotic exposure or average number of antibiotics used. Unlike several previous studies,[14–16] our series considered exposure to all antibiotics in the 8 weeks preceding symptoms. Many patients received several antibiotic regimens prior to developing diarrhea, making it difficult to identify a responsible agent. In the group of patients who had received only a single drug or regimen, we were unable to identify any antibiotic used with significantly greater frequency among the women with CDAD. Aminoglycosides, usually gentamicin, were used more commonly in the comparison group. These agents do not have an enterohepatic circulation and so do not cause significant changes in bowel flora. It is unclear if this feature contributed to the decreased frequency of CDAD among our subjects, as all patients receiving an aminoglycoside concurrently received a cephalosporin or penicillin.

The symptoms of CDAD typically develop after several days of antibiotic therapy, though they may occur as early as the first or second day or as late as several weeks after discontinuation of antibiotics.[30] Among subjects with CDAD, the onset of symptoms ranged from 4 to 35 days after initiation of antibiotics and averaged 13.7 days. Diarrhea in the comparison group usually started during or shortly after completing antibiotics. In a prior study of 151 patients with CDAD,[25] 67 (44%) had discontinued antibiotics >21 days prior to the onset of symptoms. Symptoms began in 56% of the patients after discharge from the hospital. In our series, 37% of cases developed symptoms after discharge from the hospital.

### Treatment Response

The response of CDAD to therapy, either with metronidazole or vancomycin, was good. Most patients with severe illness were treated initially with vancomycin, as has been recommended.[31] Relapse occurred in 6/23 different patients with CDAD, a frequency consistent with the experience of others.[10,17,25,32] Failure of initial therapy was equally likely after metronidazole or vancomycin. In a prior study, patients with CDAD who relapsed were of a greater age and had a higher frequency of recent abdominal surgery in comparison with patients who did not relapse.[33] No such differences were noted in the present series.

### CONCLUSIONS

CDAD is an important source of nosocomially acquired morbidity in gynecologic oncology patients. Antibiotic therapy remains the primary risk factor in the development of this infection. Individuals with a history of CDAD who again develop diarrhea are likely to be infected with *C. difficile*, and empiric therapy with metronidazole or vancomycin should be considered while the cause of the diarrhea is being determined. In addition, patients whose symptoms develop several days after completion of antibiotic therapy are at much higher risk of having CDAD than nonspecific diarrhea. Among patients who develop diarrhea while receiving antibiotics, sufficient overlap exists between those with and without *C. difficile* to make this factor clinically unreliable, though individuals with CDAD often appear more ill and suffer from worse diarrhea than individuals without *C. difficile*.

Antibiotic use is likely to maintain its significance in the medical and surgical treatment of oncology patients. A recognition of the potentially serious consequences of CDAD, including its tendency for horizontal transmission, is important. Enteric infection control procedures including isolation of infected patients and frequent hand washing are additional important measures which should be taken to minimize the development and spread of this nosocomial infection.

### ACKNOWLEDGMENTS

This work was supported by American Cancer Society Clinical Oncology Fellowship AC-37 (S.E.W.).

### REFERENCES

1. Siegel DL, Edelstein PH, Nachamkin I: Inappropriate testing for diarrheal diseases in the hospital. JAMA 263: 979–982, 1990.
2. Riley TV, Bowman RA, Carroll SM: Diarrhoea associated with *Clostridium difficile* in a hospital population. Med J Aust 166–169, 1983.
3. Aronsson B, Mollby R, Nord CE: Antimicrobial agents and *Clostridium difficile* in acute enteric disease: Epidemiological data from Sweden, 1980–1982. J Infect Dis 151:476–481, 1985.
4. Gilligan PH, McCarthy LR, Genta VM: Relative frequency of *Clostridium difficile* in patients with diarrheal disease. J Clin Microbiol 14:26–31, 1981.
5. Bartlett JG: *Clostridium difficile*: Clinical considerations. Rev Infect Dis 12(Suppl 2):S243–S251, 1990.
6. George WL: Antimicrobial agent-associated colitis and diarrhea: Historical background and clinical aspects. Rev Infect Dis 6(Suppl 1):S208–S213, 1984.
7. McFarland LV, Mulligan ME, Kwok RY, Stamm WE: Nosocomial acquisition of *Clostridium difficile* infection. N Engl J Med 320:205–210, 1989.

8. Kaatz GW, Gitlin SD, Schaberg DR, et al: Acquisition of *Clostridium difficile* from the hospital environment. Am J Epidemiol 127:1289–1294, 1988.
9. Panichi G, Pantosti A, Gentile G, et al.: *Clostridium difficile* colitis in leukemia patients. Eur J Cancer Clin Oncol 21:1159–1163, 1985.
10. Grube BJ, Heimbach DM, Marvin JA: *Clostridium difficile* diarrhea in critically ill burned patients. Arch Surg 122:655–661, 1987.
11. Heard SR, O'Farrell S, Holland D, Crook S, Barnett MJ, Tabaqchali S: The epidemiology of *Clostridium difficile* with use of a typing scheme: Nosocomial acquisition and cross-infection among immunocompromised patients. J Infect Dis 153:159–162, 1986.
12. Gerding DN, Olson MM, Peterson LR, et al.: *Clostridium difficile*-associated diarrhea and colitis in adults: A prospective case-controlled epidemiologic study. Arch Intern Med 146:95–100, 1986.
13. Cudmore MA, Silva J, Fekety R, Liepman MK, Kim KH: *Clostridium difficile* colitis associated with cancer chemotherapy. Arch Intern Med 142:333–335, 1982.
14. Thibault A, Miller MA, Gaese C: Risk factors for the development of *Clostridium difficile*-associated diarrhea during a hospital outbreak. Infect Control Hosp Epidemiol 12:345–348, 1991.
15. Brown E, Talbot GH, Axelrod P, Provencher M, Hoegg C: Risk factors for *Clostridium difficile* toxin-associated diarrhea. Infect Control Hosp Epidemiol 11:283–290,1990.
16. Zimmerman RK: Risk factors for *Clostridium difficile* cytotoxin-positive diarrhea after control for horizontal transmission. Infect Control Hosp Epidemiol 12:96–100, 1991.
17. Brunetto AL, Pearson ADJ, Craft AW, Pedler SJ: *Clostridium difficile* in an oncology unit. Arch Dis Child 63:979–981, 1988.
18. Pierce PF, Wilson R, Silva J, et al.: Antibiotic-associated pseudomembranous colitis: An epidemiologic investigation of a cluster of cases. J Infect Dis 145:269–274, 1982.
19. Kelly MT, Champagne SG, Sterlock CH, Noble MA, Freeman HJ, Smith JA: Commercial latex agglutination test for detection of *Clostridium difficile*-associated diarrhea. J Clin Microbiol 25:1244–1247, 1987.
20. Peterson LR, Olson MM, Shanholtzer CJ, Gerding DN: Results of a prospective, 18-month clinical evaluation of culture, cytotoxin testing, and culturette brand (CDT) latex testing in the diagnosis of *Clostridium difficile*-associated diarrhea. Diagn Microbiol Infec Dis 10:85–91, 1988.
21. Burdon DW, George RH, Mogg GA, et al.: Faecal toxin and severity of antibiotic-associated pseudomembranous colitis. J Clin Pathol 34:548–551, 1981.
22. Barlett JG, Taylor NS, Chang T, Dzink J: Clinical and laboratory observations in *Clostridium difficile* colitis. Am J Clin Nutr 33:2521–2526, 1980.
23. Nolan M, Kelly CP, Humphreys JF: An epidemic of pseudomembranous colitis: Importance of person to person spread. Gut 28:1467–1473, 1987.
24. Roda PI: *Clostridium difficile* colitis induced by cytarabine. Am J Clin Oncol (CCT) 10:451–452, 1987.
25. Talbot BW, Walker RC, Beart RW: Changing epidemiology, diagnosis, and treatment of *Clostridium difficile* toxin-associated colitis. Br J Surg 73:457–460, 1986.
26. Novak E, Lee JG, Seckman CE, Phillips JP, DiSanto AR: Unfavorable effect of atropine-diphenoxylate (Lomotil) therapy in lincomycin-caused diarrhea. JAMA 235: 1451–1454, 1976.
27. Mogg GAG, Keighley MR, Burdon DW, et al.: Antibiotic-associated colitis—A review of 66 cases. Br J Surg 66:738–742, 1979.
28. Gebhard RL, Gerding DN, Olson MM, et al.: Clinical and endoscopic findings in patients early in the course of *Clostridium difficile*-associated pseudomembranous colitis. Am J Med 78:45–48, 1985.
29. Block BS, Mercer LJ, Ismail MA, Moawad AH: *Clostridium difficile*-associated diarrhea follows perioperative prophylaxis with cefoxitin. Am J Obstet Gynecol 153:835–838, 1985.
30. Tedesco FJ: Pseudomembranous colitis: Pathogenesis and therapy. Med Clin North Am 66:655–664, 1982.
31. Fekety R, Shah AB: Diagnosis and treatment of *Clostridium difficile* colitis. JAMA 269:71–75, 1993.
32. Fekety R, Silva J, Kauffman C, Buggy B, Deery HG: Treatment of antibiotic-associated *Clostridium difficile* colitis with oral vancomycin: Comparison of two dosage regimens. Am J Med 86:15–19, 1989.
33. Young GP, Bayley N, Ward P, St John DJ, McDonald MI: Antibiotic-associated colitis caused by *Clostridium difficile*: Relapse and risk factors. Med J Aust 144:303–306, 1986.
